# Renal papillary calcification and the development of calcium oxalate monohydrate papillary renal calculi: a case series study

**DOI:** 10.1186/1471-2490-13-14

**Published:** 2013-03-11

**Authors:** Fèlix Grases, Antonia Costa-Bauzá, Rafel M Prieto, Antonio Conte, Antonio Servera

**Affiliations:** 1 Laboratory of Renal Lithiasis Research, Faculty of Sciences, Universitary Institute of Health Sciences Research (IUNICS), University of Balearic Islands, Palma de Mallorca, 07122, Spain

**Keywords:** Renal calculi, Pathologic calcification, Hydroxyapatite, Kidney papilla, Calcium oxalate monohydrate

## Abstract

**Background:**

The objective of this study is to determine in a case series (four patients) how calcified deposits in renal papillae are associated with the development of calcium oxalate monohydrate (COM) papillary calculi.

**Methods:**

From the recently collected papillary calculi, we evaluated retrospectively patients, subjected to retrograde ureteroscopy, with COM papillary lithiasis.

**Results:**

The COM papillary calculi were found to result from subepithelial injury. Many of these lesions underwent calcification by hydroxyapatite (HAP), with calculus morphology and the amount of HAP in the concave zone dependent on the location of the calcified injury. Most of these HAP deposits grew, eroding the epithelium covering the renal papillae, coming into contact with urine and starting the development of COM calculi. Subepithelial HAP plaques may alter the epithelium covering the papillae, resulting in the deposit of COM crystals directly onto the epithelium. Tissue calcification depends on a pre-existing injury, the continuation of this process is due to modulators and/or crystallization inhibitors deficiency.

**Conclusions:**

Since calculus morphology and the amount of detected HAP are dependent on the location and widespread of calcified injury, all types of papillary COM calculi can be found in the same patient. All patients had subepithelial calcifications, with fewer papillary calculi, demonstrating that some subepithelial calcifications did not further evolve and were reabsorbed. A high number of subepithelial calcifications increases the likelihood that some will be transformed into COM papillary calculi.

## Background

Soft tissue calcifications are a result of preexisting injury, tissue alteration and/or necrosis. The cellular detritus of the damaged extracellular matrix generated during these processes can induce calcium phosphate (hydroxyapatite [HAP]) formation through heterogeneous nucleation processes in practically all tissues because plasma and intercellular liquid are supersaturated with HAP [1]. The presence of crystallization inhibitors (non-signaling small molecules that bind to crystal nuclei or crystal faces and inhibit crystal development) can prevent or delay the formation of HAP crystals. Molecules reported to inhibit crystallization associated with soft tissue calcification include metabolic substances such as pyrophosphate [2,3], synthetic molecules including bisphosphonates [4-6], and natural products including myo-inositol hexakisphosphate (phytate) [7,8]. Cellular factors, including proteins that regulate bone mineralization, have also been implicated in the process of soft tissue calcification. The activity of such proteins can either enhance or inhibit the ability of immune system macrophages to remove HAP deposits (i.e. osteoclastic activity) [9-11]. Thus, for example, osteopontin has been shown to regulate the activity of macrophages and macrophage-derived cells (osteoclasts), facilitating phagocytosis and enhancing destruction of HAP deposits [10,11]. The combined and simultaneous action of crystallization inhibitors and the immune system can inhibit further progression and even induce the total reversion of the calcification process [9,12-14].

Papillary renal calculi are small size uroliths, mainly composed of calcium oxalate monohydrate (COM). They exhibit a typical morphology consisting of a concave face (zone of union with the papillary tissue) and an opposite smooth convex face. According to recent studies, around 13% of all renal calculi are of renal papillary type [15]. A COM papillary stone can only develop from a nidus comprised of several crystals and/or organic matter that attach to the kidney papilla. Urine remains supersaturated with calcium oxalate [16,17]. HAP has been identified as the major component of the nidus (core). In the 1930’s, Randall described a pre-calculus lesion in the renal papilla and proposed that a subepithelial calcification of renal papilla becomes the nidus of COM papillary calculi, as a consequence of the disruption of the papillary epithelial layer by the HAP plaque [18-24]. Recently, it was found that in patients susceptible to the development of calcium renal calculi, plaque is initiated in thin-loop basement membranes, basement membranes of collecting tubules, and the *vasa recta*[21-24].

This study aimed to establish relations between calcified deposits in renal papillae and the development and morphology of corresponding COM papillary renal calculi.

## Methods

### Patients and samples

From the recently collected papillary calculi, four patients with chronic stone formation requiring retrograde intrarenal surgery (RIRS) were selected. All these patients have previously spontaneously expelled some calculi, that were available for study, and none was submitted to previous urologic surgery. Their medical histories and lifestyle habits were reviewed, and their renal calculi and urine samples were obtained. The papillae of these patients were observed by flexible ureterorenoscopy. The study was a retrospective evaluation of clinical patient information. Each volunteer provided written informed consent for their clinical information to be published.

### Renal calculi

The collected stones were dried, placed in sterile containers, and examined by stereoscopic microscopy (Optomic, Madrid, Spain), infrared spectrometry (Infrared Spectroscope Bruker IFS 66, Bruker, Ettlingen, Germany), and scanning electron microscopy (Hitachi S-3400N; Hitachi, Tokyo, Japan), and microanalyzed by X-ray energy dispersion spectrometry (XFlash Detector 4010, Bruker AXS, Berlin, Germany) [25].

**Figure 1:**
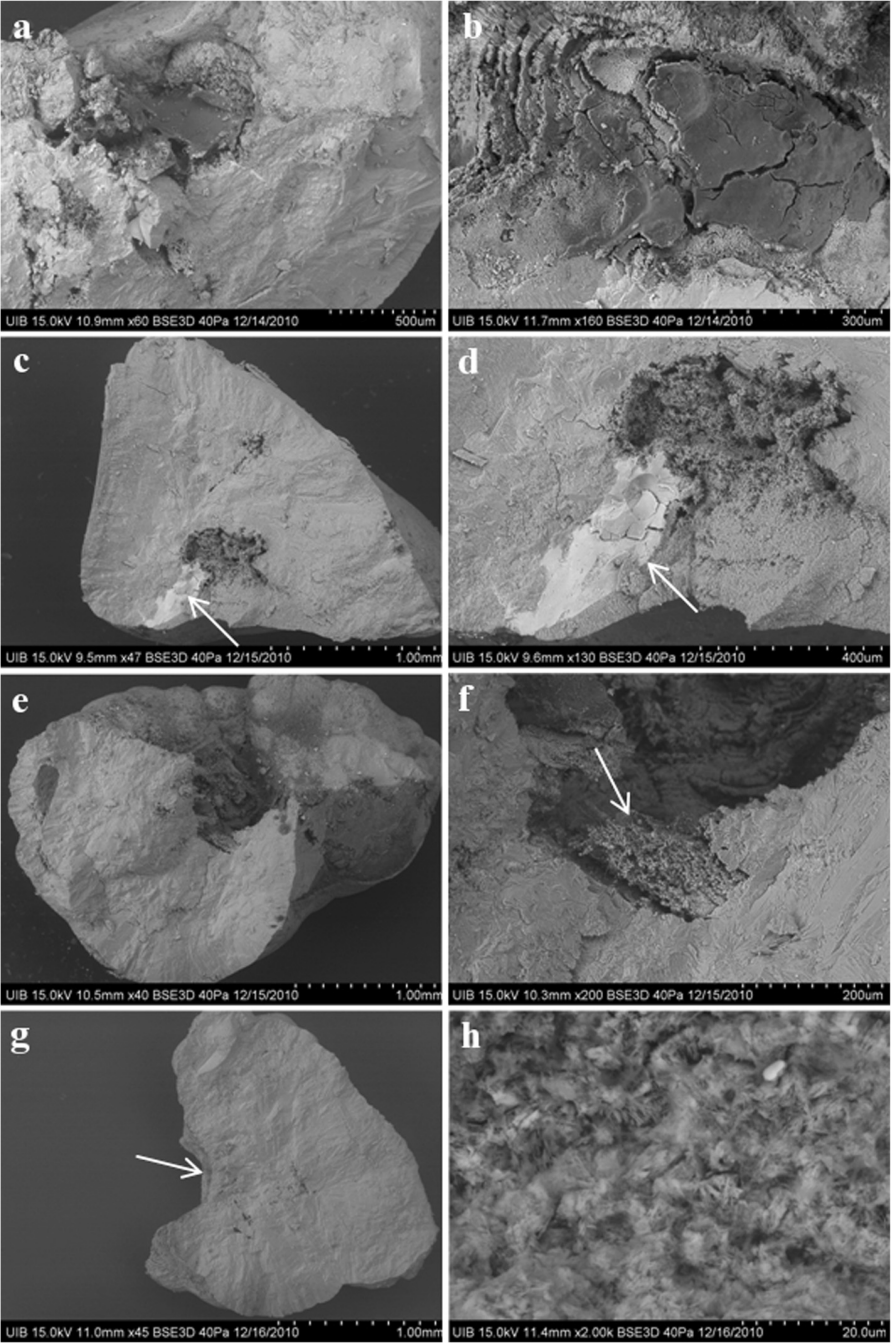
**Scanning electron microscopy images of sections of COM papillary calculi from patient 1.** (**a**, **b**) A type I calculus, in which COM columnar crystals started to develop in the concave zone in close contact with papillary tissue. (**c**, **d**) A type II calculus with a hydroxyapatite core (arrow) in or near the concave zone. (**e**, **f**) A type III calculus that developed on the papilla tip, with its concave zone containing hydroxyapatite, calcified tissue and calcified tubules (arrow). (**g**, **h**) A type IV calculus in which the core, situated near the concave zone (arrow), was formed by the intergrowth of COM crystals and organic matter.

Following direct examination of the external aspect of each stone by stereoscopic microscopy, each calculus was sectioned into two parts along a plane as near as possible to the geometric center. The internal structure and core were assessed using scanning electron microscopy and X-ray microanalysis (or IR spectrometry), which were used to identify the microcomponents present in the core and to confirm the papillary origin of these stones by examination of the concave external cavity. COM papillary calculi were identified; a typical papillary COM stone (Figure 1) consists of an eccentric core located near the concave zone, where it attaches to the papillae, and a radially striated convex peripheral layer [25]. The COM papillary calculi were classified according our classification [26] in four main types (I to IV). Type I included small calculi in which COM columnar crystals begin to develop in the concave zone in close contact with papillary tissue. Type II calculi contained a hydroxyapatite core located in or near the concave zone. Type III consisted of calculi that developed on the tip of the papillae and in the concave zone, containing hydroxyapatite, calcified tissue, and calcified tubules. Type IV consisted of papillary calculi in which the core, which is situated near, but not in, the concave zone, is formed by intergrown COM crystals and organic matter.

### Analysis of serum and urine samples

All subjects were on an unregulated diet at the time of urine collection and none of the stone-formers was receiving any pharmacological treatment. Blood was drawn from each patient, and serum was separated. Patients with renal failure or infected urine were excluded. Urine was collected over 24 hours into sterile flasks containing thymol as a preservative and immediately refrigerated. Urinary volume was recorded and the samples were stored at −20°C until analyzed. Normally, urine was collected 1–2 months after stone passage/removal.

Serum and urinary calcium, magnesium and phosphorous concentrations were measured by inductively coupled plasma atomic spectroscopy, urate and creatinine concentrations were measured using test kits (Roche Modular Analytics), and citrate and oxalate concentrations were measured using R-Biopharm enzymatic test kits. Urinary biochemical parameters were considered potential lithogenic factors under abnormal conditions.

## Results

Patient 1 was a 73-year-old woman with a 30-year history of nephrolithiasis, as well as a history of rheumatoid arthritis and arterial hypertension. She had been treated with methotrexate, furosemide and omeprazole. This patient experienced diverse renal colic episodes and spontaneously expelled 11 renal calculi, nine of which were papillary. The analysis of these calculi is summarized in Figure 1. Urinary biochemical analysis (Table 1) showed no alterations. Numerous (>3) subepithelial HAP deposits were present in the papillae of this patient.

**Table 1:** Urinary biochemical parameters in the four patients

**Urinary parameter**	**Patient 1**	**Patient 2**	**Patient 3**	**Patient 4**	**Normal range**
Volume (mL)	1300	1900	2300	1600	800-1800
Creatinine (mmols/24 h)	16.0	16.9	15.5	16.8	8.8-18.0
Calcium (mmols/24 h)	0.7	16.8	1.9	7.6	2.5-7.5
Magnesium (mmols/24 h)	2.9	7.9	3.0	4.1	3-5
Phosphorus (mmols/24 h)	15	36	23	42	12-39
Oxalate (mmols/24 h)	0.40	0.43	0.42	0.30	0.04-0.50
Urate (μmols/24 h)	2.1	3.9	3.1	3.7	1.5-3.5
Citrate (mmols/24 h)	3.5	2.9	1.9	6.18	1.5-4.9

Of the nine papillary calculi retrieved from patient 1, six had HAP deposits located in or near the concave zone of the calculus (Figure 1c, 1d). These calculi were associated with papillary lateral extruded HAP deposits, indicating that they were type II papillary calculi [26]. Another two calculi were associated with lateral subepithelial HAP deposits (Figure 1a, 1b), corresponding to type I [26]. One calculus developed on the tip of the papillae (Figure 1e, 1f) and corresponded to type III [26]. Finally, one calculus had a core, located near the concave zone, formed by a combination of COM crystals and organic matter (Figure 1g, 1h), corresponding to a type IV papillary calculus [26].

**Figure 2:**
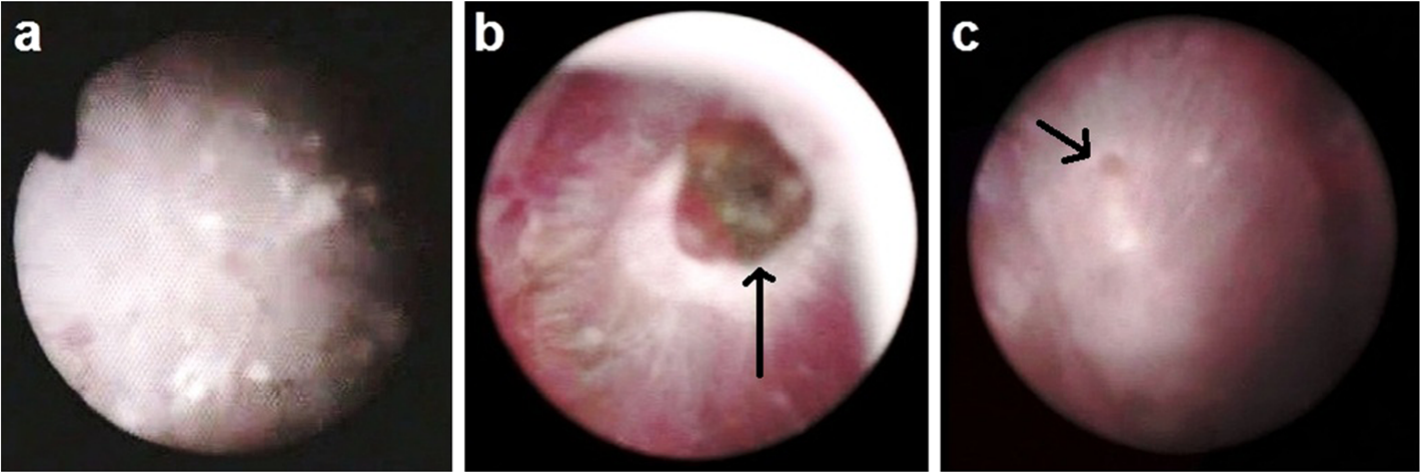
**Hydroxyapatite deposits (endoscopic images) of renal papillae from patients (a) 2, (b) 3, and (c) 4.** A papillary calculus was present on the papillary tip in patient 3 (arrow), and a small papillary calculus was present at the lateral position in patient 4 (arrow).

Patient 2 was a 40-year-old man with a history of nephrolithiasis. He had no other pathology or health problem of significance. This patient had consumed a hyperproteic diet (very rich in meat) and spontaneously expelled a typical calculus of calcium oxalate dihydrate. Urinary biochemical analysis (Table 1) showed that this patient had hypercalciuria and high oxaluria. Endoscopic images of his papillae showed numerous (> 3) subepithelial HAP deposits (Figure 2a).

**Figure 3:**
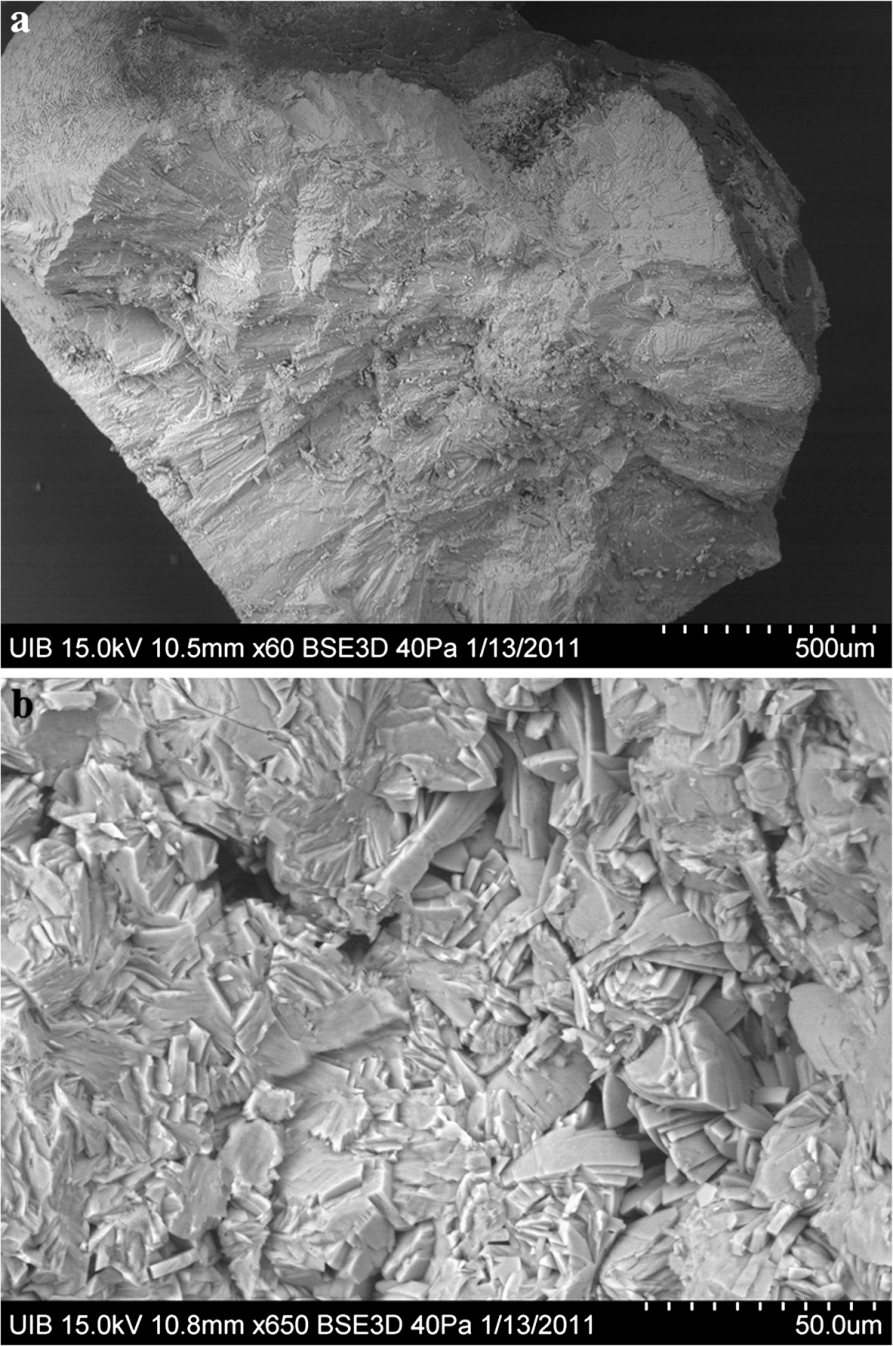
**Scanning electron microscopy images of a section of a COM papillary calculus from patient 3.** (**a**) General view of the calculus section. (**b**) Calculus core formed by the intergrowth of COM crystals and organic matter.

Patient 3 was a 60-year-old man with a history of nephrolithiasis. He had no other pathology or health problem of significance. He spontaneously expelled a COM papillary calculus, corresponding to type IV (Figure 3). Urinary biochemical analysis (Table 1) showed high oxaluria but abnormally low calciuria (1.5 mmol/L). Endoscopic images of his papillae showed two attached calculi, one at the top and the other in a lateral position (Figure 2b). Moreover, these papillae showed numerous (> 3) subepithelial HAP deposits.

Patient 4 was a 41-year-old man with nephrolithiasis and hyperuricemia, but no other pathology or significant health problem. The patient spontaneously expelled a typical calcium oxalate dihydrate calculus, partially transformed into COM. Urinary biochemical analysis (Table 1) showed that this patient had hypercalciuria and hyperphosphaturia. Endoscopic images showed a small COM calculus attached to one side of the papillae (Figure 2c). In addition, the papillae of this patient showed numerous (> 3) subepithelial HAP deposits.

## Discussion

The results in our patients indicate that COM papillary calculi result from subepithelial lesions in the tip of the papilla, emerging in a renal calyx. Most lesions undergo calcification by HAP; these deposits grow and erode the epithelium covering the papillae. When these deposits come into contact with urine, they start the development of a COM calculus due to the permanent supersaturation of calcium oxalate in urine and to the high capacity of HAP to act as a heterogeneous nucleant of COM. These calcifications are generated in the thin-loop basement membranes or near the *vasa recta*[27,28], both of which are collagen-rich regions [28]. Due to the presence of carboxylate groups, this altered collagen can act as a heterogeneous nucleant of HAP [29].

The concave zone of papillary calculi can contain a range of calcified lesions, from little HAP residues to typical well-developed HAP plaques, also known as Randall's plaques. The morphology of each calculus and the amount of detected HAP will depend on both the calculus and the location of each calcified injury. HAP plaques may be located in a subepithelial position, altering the epithelium covering the papillae and resulting in the direct deposit of COM crystals on the damaged epithelium [27]. Less frequently, the injury may be in close contact with the epithelium covering the papillae; expansion of the injury may alter the epithelium, allowing the nucleation of COM crystals. These COM calculi will not contain HAP in their starting zone.

These results indicate that COM papillary calculi might be generated from subepithelial lesions on the tip of the renal papillae, and that the morphology of these COM calculi mainly depends on the location of the injury. Hence, the examination of a single calculus does not supply relevant information about the origin of the injury and the severity of the general calculogenesis process. The possible causes of injury may be deduced from the clinical data and lifestyle of each patient [26]. Thus, the papillary injuries in patient 1 may be due to the chronic consumption of drugs to treat her rheumatoid arthritis, as well as to arterial hypertension. The papillary tissue injuries in patients 2 and 3 may have been due to high urinary oxalate, and the injury in patient 4 may have been due to hyperuricemia observed on the clinical history of the patient.

Dystrophic mineralization is commonly observed in soft tissues as a result of injury. Although most soft tissues can undergo calcification, kidney, tendons, and cardiovascular tissues appear particularly prone to developing this pathology [30]. Although the development of tissue calcification depends on a pre-existing injury, which acts as an inducer, the continuation of this process is due to modulators and/or a deficiency in crystallization inhibitors [31-37]. For example, some carboxy proteins, such as osteopontin, can bind to HAP, recruiting macrophages that remove these calcifications or prevent their progression [31-36]. The inhibition of crystallization, preventing HAP development (nucleation and growth), may be due to low-molecular weight-compounds, such as pyrophosphate, magnesium and phytate [2,37,38]. The images of the papillae in our patients suggest that many subepithelial calcifications were present in all four, although the number of papillary calculi generated were substantially lower, suggesting that some of these subepithelial calcifications did not progress further and were reabsorbed. An evaluation of Randall’s plaque theory of nephrolithiasis in patients with a unilateral single papillary calculus showed that computed tomography attenuation values (Hounsfield units) of all papillae on the affected side were significantly greater in patients with stone formation than in those without stone formation [39]. In contrast, no significant differences were found between the affected and non-affected sides of patients with stone formation [39], this demonstrating that not all internal calcification progress further to forming stones.

## Conclusions

Although tissue calcification depends on a pre-existing injury, the continuation of this process is due to modulators and/or crystallization inhibitors deficiency. Since calculus morphology and the amount of detected HAP are dependent on the location and widespread of calcified injury, all types of papillary COM calculi can be found in the same patient. All patients had large numbers of subepithelial calcifications, with fewer papillary calculi, demonstrating that some subepithelial calcifications did not further evolve and were reabsorbed. A high number of subepithelial calcifications increases the likelihood that some will be transformed into COM papillary calculi. Thus, when papillary COM calculi are identified, the main objective to avoid recurrent episodes, should be the identification and elimination of the cause that originates the intrapapillary injury (since in such cases it is the main cause of stone development). Obviously, an adequate level of crystallization inhibitors and the proper activity of the immune system can favorably contribute to avoid the development of these calculi. As an important limitation of this study, it must be considered that a series of only four patients were included due the difficulty to find patients with the required inclusion criteria.

### Informed consent

Written informed consent was obtained from patients for publication of this retrospective study.

## Competing interests

The authors declare that they have no competing interests.

## Authors’ contributions

FG participated in the design, evaluation and discussion of the obtained results and coordination. AC-B participated in examination of samples and evaluation and discussion of the obtained results. RMP participated in the obtention of biochemical information and image processing and evaluation of obtained results AC participated in the obtention of biological samples and evaluation of obtained results AS participated in the obtention of biological samples and image processing. All authors read and approved the final manuscript.

## Pre-publication history

The pre-publication history for this paper can be accessed here:

http://www.biomedcentral.com/1471-2490/13/14/prepub

## References

[B1] Epple M Lanzer P How much interdisciplinarity is required to understand vascular calcifications? Formulation of four basic principles of vascular calcification Z Kardiol 2001 90 Suppl III 2 5 11374027 10.1007/s003920170036

[B2] Lomashvili KA Cobbs S Hennigar RA Hardcastle KI O'Neill WC Phosphate-induced vascular calcification, role of pyrophosphate and osteopontin J Am Soc Nephrol 2004 15 1392 1401 10.1097/01.ASN.0000128955.83129.9C 15153550

[B3] Fleisch H Bisaz S Mechanism of calcification, inhibitory role of pyrophosphate Nature 1962 195 911 13893487 10.1038/195911a0

[B4] Bevilacqua M Dominguez LJ Rosini S Barbagallo M Bisphosphonates and atherosclerosis, why? Lupus 2005 14 773 779 10.1191/0961203305lu2219oa 16218486

[B5] Price PA Faus SA Williamson MK Bisphosphonates alendronate and ibandronate inhibit artery calcification at doses comparable to those that inhibit bone resorption Arterioscler Thromb Vasc Biol 2001 21 817 824 10.1161/01.ATV.21.5.817 11348880

[B6] Price PA Buckley JR Williamson MK The amino bisphosphonate ibandronate prevents vitamin D toxicity and inhibits vitamin D-induced calcification of arteries, cartilage, lungs and kidneys in rats J Nutr 2001 131 2910 2915 11694617 10.1093/jn/131.11.2910

[B7] Grases F Sanchis P Perello J Isern B Prieto RM Fernandez-Palomeque C Fiol M Bonnin O Torres JJ Phytate (myo-inositol hexakisphosphate) inhibits cardiovascular calcifications in rats Front Biosci 2006 11 136 142 10.2741/1786 16146720

[B8] Grases F Sanchis P Perelló J Isern B Prieto RM Fernández-Palomeque C Torres JJ Effect of crystallization inhibitors on vascular calcifications induced by vitamin D, A Pilot Study in Sprague–Dawley Rats Circ J 2007 71 1152 1156 10.1253/circj.71.1152 17587727

[B9] Bas A Lopez I Perez J Rodriguez M Aguilera-Tejero E Reversibility of calcitriol-induced medial artery calcification in rats with intact renal function J Bone Miner Res 2006 21 484 490 16491297 10.1359/JBMR.051211

[B10] Redey SA Razzouk S Rey C Bernache-Assollant D Leroy G Nardin M Cournot G Osteoclast adhesion and activity on synthetic hydroxyapatite, carbonated hydroxyapatite, and natural calcium carbonate, relationship to surface energies J Biomed Mater Res 1999 45 140 147 10.1002/(SICI)1097-4636(199905)45:2<140::AID-JBM9>3.0.CO;2-I 10397968

[B11] Shen M Marie P Farge D Carpentier S De Pollak C Hott M Chen L Martinet B Carpentier A Osteopontin is associated with bioprosthetic heart valve calcification in humans C R Acad Sci III 1997 320 49 57 10.1016/S0764-4469(99)80086-9 9099263

[B12] Najman S Dordevic L Savic V Ignjatovic N Plavsic M Uskokovic D Changes of HAp/PLLA biocomposites and tissue reaction after subcutaneous implantation Facta Universitatis 2003 10 131 134

[B13] Nadra I Mason JC Philippidis P Florey O Smythe CDW McCarthy GM Landis RC Haskard DO Proinflammatory activation of macrophages by basic calcium phosphate crystals via protein kinase C and MAP kinase pathways, a vicious cycle of inflammation and arterial calcification? Circ Res 2005 96 1248 1256 10.1161/01.RES.0000171451.88616.c2 15905460

[B14] Sun YB Zeng XR Wenger L Cheung HS Basic calcium phosphate crystals stimulate the endocytotic activity of cells-inhibition by anti-calcification agents Biochem Biophys Res Commun 2003 312 1053 1059 10.1016/j.bbrc.2003.11.048 14651978

[B15] Grases F Costa-Bauzá A Ramis M Montesinos V Conte A Simple classification of renal calculi closely related to their micromorphology and etiology Clin Chim Acta 2002 22 29 36 12104078 10.1016/s0009-8981(02)00063-3

[B16] Finlayson B Physicochemical aspects of urolithiasis Kidney Int 1978 13 344 360 10.1038/ki.1978.53 351263

[B17] Robertson WG Peacock M Nordin BEC Activity products in stone-forming and non-stone-forming urine Clin Sci 1968 34 579 594 5666884

[B18] Randall A Origin and growth of renal calculi Ann Surg 1937 105 1009 1027 10.1097/00000658-193706000-00014 17856988 PMC1390483

[B19] Prien EL The riddle of Randall's plaques J Urol 1975 114 500 507 1235369 10.1016/s0022-5347(17)67068-x

[B20] Low RK Stoller ML ML Endoscopic mapping of renal papillae for Randall’s plaques in patients with urinary stone disease J Urol 1997 158 2062 2064 10.1016/S0022-5347(01)68153-9 9366312

[B21] Evan AP Lingeman JE Coe FL Parks JH Bledsoe SB Shao Y Sommer AJ Paterson RF Kuo RL Grynpas M Randall's plaque of patients with nephrolithiasis begins in basement membranes of thin loops of Henle J Clin Invest 2003 111 607 616 12618515 10.1172/JCI17038 PMC151900

[B22] Kim SC Coe FL Tinmouth WW Kuo RL Paterson RF Parks JH Munch LC Evan AP Lingeman JE Stone formation is proportional to papillary surface coverage by Randall's plaque J Urol 2005 173 117 119 10.1097/01.ju.0000147270.68481.ce 15592050

[B23] O’connor RC Worcester EM Evan AP Meehan S Kuznetsov D Laven B Sommer AJ Bledsoe SB Parks JH Coe FL Grynpas M Gerber GS Nephrolithiasis and nephrocalcinosis in rats with small bowel resection Urol Res 2005 33 105 115 10.1007/s00240-004-0460-4 15815943

[B24] Evan AP Coe FL Rittling SR Bledsoe SM Shao T Lingeman JE Worcester EM Apatite plaque particles in inner medulla ofkidneys of calcium oxalate stone formers: osteopontin localization Kidney Int 2005 68 145 154 10.1111/j.1523-1755.2005.00388.x 15954903

[B25] Grases F García-Ferragut L Costa-Bauzá A Analytical study of renal calculi. A new insight Rec Res Dev Pure Appl Anal Chem 1998 1 187 206

[B26] Grases F Costa-Bauzá A Gomila I Conte A Origin and types of calcium oxalate monohydrate papillary renal calculi Urology 2010 76 1339 1345 10.1016/j.urology.2010.02.022 20466410

[B27] Evan AP Coe FL Lingeman JE Shao Y Sommer AJ Bledsoe SB Anderson JC Worcester EM Mechanism of formation of human calcium oxalate renal stones on Randall’s plaque Anat Rec 2007 290 1315 1323 10.1002/ar.20580 17724713

[B28] Evan AP Weinman EJ Wu XR Lingeman JE Worcester EM Coe FL Comparison of the pathology of interstitial plaque in human ICSF stone patients to NHERF-1 and THP-null mice Urol Res 2010 38 439 452 10.1007/s00240-010-0330-1 21063698 PMC3035321

[B29] Takeuchi A Ohtsuki C Miyazaki T Kamitakahara M Ogata S Yamazaki M Furutani Y Kinoshita H Tanihara M Heterogeneous nucleation of hydroxyapatite on protein, structural effect of silk sericin J R Soc Interface 2005 2 373 378 10.1098/rsif.2005.0052 16849195 PMC1578267

[B30] Anderson HC Morris DC Mundy GR, Martin TJ Mineralization Physiology and Pharmacology of Bone 1993 New York: Springer Verlag 267 298

[B31] Steitz SA Speer MY McKee MD Liaw L Almeida M Yang H Giachelli CM Osteopontin inhibits mineral deposition and promotes regression of ectopic calcification Am J Pathol 2002 161 2035 2046 10.1016/S0002-9440(10)64482-3 12466120 PMC1850905

[B32] Romberg RW Werness PG Riggs BL Mann KG Inhibition of hydroxyapatite crystal growth by bone-specific and other calcium binding proteins Biochemistry 1986 25 1176 1180 10.1021/bi00353a035 3008822

[B33] Boskey AL Maresca M Ullrich W Doty SD Butler WT Prince CW Osteopontin-hydroxyapatite interactions in vitro: inhibition of hydroxyapatite formation and growth in a gelatin-gel Bone Miner 1993 22 147 159 10.1016/S0169-6009(08)80225-5 8251766

[B34] Govindaraj A Selvam R An oxalate-binding protein with crystal growth promoter activity from human kidney stone matrix BJU Int 2002 90 336 344 10.1046/j.1464-410X.2002.02849.x 12133075

[B35] Yamate Y Kohri K Umekawa T Amasaki N Amasaki N Isikawa Y Iguchi M Kurita T The effect of osteopontin on the adhesion of calcium oxalate crystals to Madin-Darby canine kidney cells Eur Urol 1996 30 388 393 8931975 10.1159/000474201

[B36] Lieske JC Toback FG Deganello S Sialic acid-containing glycoproteins on renal cells determine nucleation of calcium oxalate dihydrate crystals Kidney Int 2001 60 1784 1791 10.1046/j.1523-1755.2001.00015.x 11703596

[B37] Grases F Isern B Sanchis P Perello J Torres JJ Costa-Bauza A Phytate acts as an inhibitor in formation of renal calculi Front Biosci 2007 12 2580 2587 10.2741/2256 17127264

[B38] Wilson JW Werness PG Smith LH Inhibitors of crystal growth of hydroxyapatite, a constant composition approach J Urol 1985 134 1255 1258 2997488 10.1016/s0022-5347(17)47706-8

[B39] Bhuskute NM Yap WW Wah TM A retrospective evaluation of Randall’s plaque theory of nephrolithiasis with CT attenuation values Eur J Radiol 2009 72 470 472 10.1016/j.ejrad.2008.09.009 18947952

